# Fungal brain infection—no longer a death sentence

**DOI:** 10.1007/s10143-020-01410-3

**Published:** 2020-10-09

**Authors:** Nicole Lange, Nina Wantia, Ann-Kathrin Jörger, Arthur Wagner, Friederike Liesche, Bernhard Meyer, Jens Gempt

**Affiliations:** 1 Department of Neurosurgery, Klinikum rechts der Isar, Technical University Munich, Munich, Germany; 2 Department of Microbiology, Klinikum rechts der Isar, Technical University Munich, Munich, Germany; 3 Department of Neuropathology, Klinikum rechts der Isar, Technical University Munich, Munich, Germany

**Keywords:** Aspergillosis, Cerebral, Outcome, Risk factors

## Abstract

The aim of this case series was to provide a modern cohort of patients with cerebral aspergillosis and show the effectiveness of modern treatment concepts. In a 10-year period from January 2009 to January 2019, we identified 10 patients (6 male, 4 female) who received surgery or frameless stereotactic drainage of a cerebral aspergilloma at our center. Patients’ and disease characteristics were recorded. The median age was 65 (range 45 to 83). We conducted 133 cranial surgeries in 100 patients due to cerebral brain abscess (BA) during that time, which leads to a percentage of 10% of aspergilloma within BAs in our patient sample. We performed 3.1 surgeries per patient followed by antifungal treatment for 6 months (= median) according to the microbiological findings. Regarding comorbidities, the mean Charlson comorbidity index (CCI) at the time of admission was 5, representing an estimated 10-year survival of 21%. Six (60%) of 10 patients showed conditions of immunosuppression, one suffered endocarditis after replacement of aortic valves. Four patients showed associated frontobasal bone destruction, mycotic aneurysms, or thromboses. The mean duration of hospital stay was 37 days. Mortality was much lower than in literature. Sixty percent of the patients died during the follow-up period. The outcome of the two immunocompetent patients was more favorable. Cerebral aspergillosis is a rare, but still life-threatening, condition, which predominantly occurs in immunosuppressive conditions. Due to radical surgical and antifungal therapy for several months, mortality can be reduced dramatically.

## Introduction

Aspergillosis is a rare type of infection caused by *Aspergillus fumigatus*, an ubiquitous mold (a fungal organism) of which spores are an inherent component of breathing air [3]. It primarily leads to infection in patients with underlying immunosuppression, such as autoimmune diseases, hematopoietic stem cell or solid organ transplantations, or tumor diseases. Incidence estimates of 1992 in the USA suggested a yearly rate of two cases of aspergillosis per 100,000 population [34]. *Aspergillus fumigatus* is the most common species in human infections. Once the central nervous system (CNS) is infected, the prognosis is poor [10, 37]. Thereby, brain involvement was only found in 3% of all patients with aspergillosis, and in 20% and up to 42% within the subgroups of previously diagnosed tumor disease, or patients with acute leukemia and hematopoietic stem cell transplantation [4, 19, 28, 40]. Brain involvement most commonly results in brain abscess formation, occasionally accompanied by cerebral infarction due to septic embolisms, or associated mycotic aneurysms [5, 17, 18, 21, 25, 28]. Prognosis of cerebral aspergillosis is poor. A literature review of 1996 revealed a mortality rate of 99% within 141 patients of this particular type of intracerebral mold infection [11]. In 2001, the case fatality rate (CFR) was estimated 88% [23]. Literature shows that therapy concepts of successfully treated aspergillomas contain radical surgical excision of affected tissue and long-term antimycotic therapy [9, 20]. More recent data of patients with cerebral aspergilloma is rare and mostly within case reports.

*Pseudallescheria boydii*, its asexual form known as *Scedosporium apiospermum*, is a fungus belonging to the Ascomycota. It is typically found in water and is an emerging opportunistic pathogen. It is the second most common fungus after *Aspergillus fumigatus* found in patients with cystic fibrosis. In immunocompromised hosts, infection can occur in the lung, skin, bones, and CNS. CNS infection is a very rare incident, and the prognosis is poor [27].

The aim of this case series was to provide a modern cohort of patients with cerebral aspergillosis, assess the clinical characteristics, and reveal the relevant aspects for future therapy for such a condition. Furthermore, we aimed to show the effectiveness of our treatment by analyzing our patients’ clinical outcomes and to identify some risk factors for a poor outcome of cerebral aspergillosis.

## Materials and methods

### Patient population

We identified all patients receiving surgery or frameless stereotactic drainage of a cerebral aspergilloma in our center in a 10-year period from January 2009 to January 2019. We included patients with suspect lesions in preoperative MRI or CT scans, which showed isolated fungi of the genus *Aspergillus fumigatus* from the brain tissue and histopathologically proven signs of fungal infection. The present study was approved by the local ethics committee and performed in accordance with the ethical standards established by the 1964 Declaration of Helsinki and its later amendments [39] (Clinical Trial Registration Number: 217/16S).

Patients’ and disease characteristics were recorded, including age, mortality, dates and types of surgical procedures, neurological symptoms, and length and type of antifungal treatment. Germ spectrum and immunosuppressive conditions were also assessed.

### Outcome

For all patients, the CCI [7] was calculated at the time of admission. The clinical parameters were the Barthel index (BI) [36] at the time of admission and after surgery, as well as the modified Rankin scale (MRS) [32] at the time of admission, discharge, and several times during the follow-up period (3 months, 1 year, 2 years, and 5 years). Data regarding the antifungal treatment, germ spectrum, and BI was taken from our database. As a retrospective analysis, patients’ informed consent was not necessary.

### Statistical data analysis

Statistical analyses, including descriptive data analyses, were performed using IBM SPSS Statistics version 22.0 (IBM Corporation, New York). Associations between all assessed variables were analyzed using chi-square tests. To identify possible risk factors for outcome changes, logistic regression analysis was done. For all analyses, a difference with an error probability of less than 0.05 was considered statistically significant. Descriptive statistics for demographic variables were generated with means and SDs or medians with interquartile ranges as appropriate.

## Results

Clinical characteristics of the patients are presented in Table 1.

**Table 1:** Demographic and clinical characteristics of patients

Age (years; mean, range)	65 (45–83)
Sex (*N* =)	
Female	6
Male	4
CCI (points; mean, SD)	4.8 (2.8)
Immunosuppression (%)	60
B cell lymphoma	17
Renal cell carcinoma	17
Hematopoietic stem cell transplant	17
Long-term cortisone intake	17
B ALL	17
Tolosa-Hunt syndrome	17
Symptoms (%)	
Paresis	40
Visual impairment	30
Headache	30
Cranial nerve paresis	20
Vigilance decrement	20
Fever	20
Aphasia	10
Gait disturbance	10
Loss of fine motor skills	10
Loss of olfactory sense	10
Incidental findings	10

In a 10-year period from January 2009 to January 2019, we identified 10 patients (6 male, 4 female) who received surgery or frameless stereotactic drainage of a cerebral aspergilloma at our center. We conducted 133 cranial surgeries in 100 patients due to cerebral brain abscess (BA) during that time, which leads to a percentage of 10% of aspergilloma within BAs in our patient sample.

The mean age was 65 years (range 45–83).

Regarding comorbidities, mean CCI at the time of admission was 4.8 representing an estimated 10-year survival of 21% [8]. Six (60%) of 10 patients showed conditions of immunosuppression. Those are presented in Table 2 (B cell lymphoma, renal cell carcinoma, hematopoietic stem cell transplantation, long-term cortisone because of glomerulonephritis, B ALL, and Tolosa-Hunt syndrome). Of the remaining four patients, one suffered severe endocarditis and underwent surgical replacement of aortic and tricuspid valves 1 month before septic cerebral aspergillosis was detected. Relevant comorbidities of this patient were diabetes and COPD. Another patient suffered severe sepsis due to perforation of sigmoid diverticulitis with multiple abdominal operations 3 months before the development of brain abscess. Two patients seemed to be immunocompetent. Further, 5 patients (50%) showed solitaire abscesses, and in 5 patients (50%) multiple BAs could be identified.

**Table 2:** Operative and clinical details of patient collective

Patient no.	Sex	Age	Death	CCI at admission	Immunosuppression	Location	Associated pathology	Count of surgeries
1	m	80	†	11	y	Ethmoidal cells, sinus cavernosus, sinus sphenoidalis	Mycotic aneurysm anterior communicating artery	6
2	f	50	–	3	y	Left operculum		2
3	f	83	–	4	n	Sinus maxillaris und bifrontal	Frontobasal destruction	5
4	m	56	†	4	y	Bifrontal, right side occipital	Thrombosis of ACI	3
5	m	74	†	6	n	Sinus maxillaris, sella turcica	Thrombosis of ACI	1
6	m	58	†	7	y	Left frontal, right central region		1
7	f	71	–	6	y	Fissura orbitalis superior right, sinus sphenoidalis, ethmoidal cells		1
8	m	58	†	2	y	Both sided occipital, right temporal, left cerebellar		3
9	m	73	–	3	y	Both sided frontal, sphenoidal sinus		6
10	f	45	†	2	y	right parietal, left frontal		3

Symptoms leading to diagnosis were of wide range: paresis in 40%, visual impairment and headache in 30%, paresis of cranial nerves (oculomotorius), vigilance decrement, and fever in 20% and in 10%, aphasia, disturbances in fine motor skills, loss of olfactory sense, gait disorders, and incidental findings on CT scans.

Infection parameters, measured by C reactive protein (CRP; standard values < 0.5 mg/dl) levels at the time of admission, were elevated in most of the patients (70%), showing mean CRP levels of 1.6 (SD 1.6).

### Surgical procedures

In total, we performed 31 surgical procedures (3.1 per patient). Abscess evacuations (23 surgeries) were conducted via open craniotomies in 14 surgeries (61%), VarioGuide aspiration in 5 (22%), transnasal in 4 (17%). Additional four surgeries were performed for treatment of aspergilloma-associated pathologies (2 bypass surgeries for associated aneurysms, 2 frontobasal reconstructions for persisting frontobasal defects). The remaining four procedures were due to complications (3 evacuations of postoperative hematomas, 1 ventriculoperitoneal-shunt-implantation because of hydrocephalus malresorptivus). In 4 cases, craniotomy was chosen because frontobasal reconstruction was needed in addition to abscess evacuation. One case required a split bone graft.

One patient needed endovascular thrombectomy and stenting due to septic embolism of ACI. Initial surgical procedures and patient presentations were emergency procedures because of rapid deterioration of the patients in 80%.

There were no cases of operative mortality.

### Microbiological findings

Analysis of the abscess tissue showed *Aspergillus fumigatus* in nine cases and *Pseudallescheria boydii* in one case. Four patients showed additional germs (*E. coli*, *Staph aureus*, *herpes simplex virus*). In our patient samples, diagnosis was made through microscopy and culture in 6 patients. Four samples were analyzed via PCR in addition.

In every patient, focus identification was conducted via the consultation of cardiologists (including transesophageal echocardiography); gynecologists or urologists; oral and maxillofacial surgeons; ear, nose, and throat (ENT) physicians; thoracic X-ray; and urine analysis. The focus for aspergilloma was the former surgical site in 3 cases and unknown in 7 cases.

Treatment was with voriconazole (azole antimycotic) in 6 cases, voriconazole and amphotericin B (streptomycetes) in 3 cases, and in one case with caspofungin (echinocandin). Additional virostatic medication was given in one patient and antibiotics in two patients. The mean duration of treatment was 5.8 months (SD 5.2).

Analysis of blood cultures was done in all cases and turned out to be negative.

A reliable diagnosis of fungal infection is challenging.

Both microscopy and culture should be attempted on appropriate specimens from patients at risk for IA (AII) with a priority for culture in most cases where insufficient material is available. Demonstrating tissue invasion by hyphae through microscopic examination of biopsy or autopsy material provides a diagnosis of proven invasive fungal infection. However, the sensitivity of microscopy for IA is 50% at best [14, 35].

PCR approach can increase the sensitivity to 57–> 90% depending on the amount of fungi in the sample. The culture and subculture of fungi contain Sabouraud dextrose agar; identification is performed by microscopy or sequencing of the fungus. Isolates are screened for azole resistance using supplemented plates with voriconazole and itraconazole. All isolates of *Aspergillus fumigatus* were azole susceptible. The isolate of *Pseudallescheria boydii* is known to be amphotericin-resistant; the patient was treated with voriconazole.

### Outcome

**Fig. 1:**
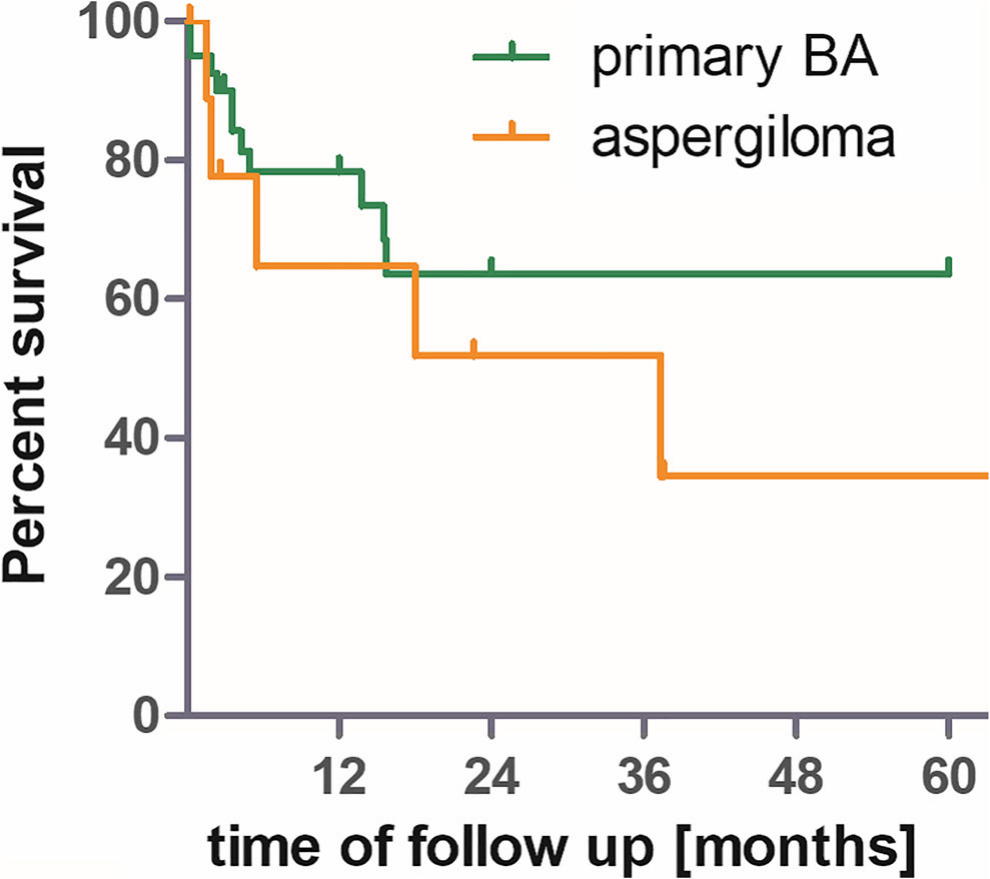
Survival curves of our patient collective. The patients with primary brain abscess due to bacteria are treated in our center during the same period. Log-rank test shows no significant differences in survival curves: HR 0.43, 95% CI (0.1053–1.735)

Figure 1 shows survival curves of our patient collective and the patients with primary brain abscess due to bacteria are treated in our center during the same period. Log-rank test shows no significant differences in survival curves: HR 0.43, 95% CI (0.1053–1.735).

The overall mortality was 60%. Half of the patients died during the first 2 months of treatment. Deaths were all associated with aspergilloma, leading to septic embolisms and multi-organ failure in most cases. All patients could be followed, so there were no patients lost to follow-up.

The mean MRS at the time of admission, discharge, and after 1 year was 3, 4, and 5, respectively. The mean length of hospital stay was 37 days.

## Discussion

In this analysis of 10 patients, treated at our center between 2009 and 2019 for cerebral aspergillosis, we assessed clinical characteristics, outcomes, and survival rates of this patient collective.

Cerebral aspergillosis stays a very rare condition. We conducted 133 cranial surgeries in 100 patients due to cerebral brain abscess (BA) during that time, which leads to a percentage of 10% of aspergilloma within BAs in our patient sample.

Regarding comorbidities, the mean CCI at the time of admission was 5, representing an estimated 10-year survival of 21% [8]. Six (60%) of 10 patients showed conditions of immunosuppression. This is in good accordance with the literature, which describes disseminated aspergillosis and CSF infection much more common in an immunocompromised host than in healthy persons [26]. It is seen as an opportunistic infection in AIDS, but also in organ transplantation patients, in neutropenia associated with acute leukemia and chemotherapy, and after long-term steroid therapy [15]. In our patient collective, two patients seemed to be immunocompetent, without any preexisting conditions favoring fungal infections. Abscesses were both located in the maxillar sinus and lead to frontobasal destruction invading the frontal lobe. They both survived after surgical resection and long-term antimycotic therapy. Thus, the mechanisms for those infections stay unclear, the number of patients seems to be rising, while prognosis in those patients is more favorable [6, 12].

The overall mortality of patients with cerebral fungal infection was 60%. During the same period, we analyzed all patients with primary brain abscesses treated in our center. In that collective, 23% died during the follow-up period [21]. This underlines an infection with fungi as a still very severe condition, even though log-rank test could not show a significant difference due to the small sample size. In the literature, mortality with fungal brain abscess ranges between 80 and 99%. This shows that modern treatment strategies as shown in this case series, comprising radical operative resections followed by long-term antimycotic therapies, are able to reduce mortality in this modern era dramatically [1, 11, 23]. Of course, this reduction of mortality can not only be ascribed to radical surgical removal of affected tissue but also to the development of new antifungal therapy as voriconazole. Taking into account some case reports describing patients recovering with long-term antifungal therapy and surgical aspirations only but on the other hand descriptions of voriconazole-refractory invasive aspergillosis, requiring radical surgical resections and even lobectomies, therapy concepts need to be more individual and patient specific [2, 16, 24, 29]. Reduction of infection via stereotactic aspiration or open surgical removal is required in every case. In this collective, every patient with stereotactic biopsy had to undergo open surgical removal of abscess tissue some weeks later due to progression in follow-up MRI despite voriconazole therapy. Nowadays, radical excision of abscess tissue is a safe procedure, leaving patients in a good clinical condition which they need for a long antifungal treatment with many side effects [22]. Finally, diagnosis can be placed fast and earlier due to new imaging techniques and their broad availability.

Histopathologically, single or multiple abscess formations with invasion of blood vessels leading to thromboses, septic embolisms, or mycotic aneurysms are characteristics of aspergillosis [13, 30, 31]. In accordance with this, two of our patients suffered thromboses of internal carotid artery (one needed thromboendarterectomy): one needed bypass surgery for a huge mycotic aneurysm of the anterior communicating artery. The outcome of those patients was fatal.

Aspergillosis usually manifests with acute onset of focal neurologic deficits, confirming that 80% of initial surgical procedures were emergency procedures because of rapid deterioration of the patients. Symptoms are often of wide range, as described previously [33, 38]. Interestingly, one of our patients showed no symptoms at all. Multiple aspergillomas were detected during routine staging. He suffered neutropenia because of ALL and following chemotherapy. Routine surgery was planned, while 1 day prior to surgery date, he developed hemiparesis.

## Conclusion

Cerebral aspergillosis is a rare, but still life-threatening, condition, which predominantly occurs in immunosuppressive conditions. Due to radical surgical removal of infected tissue and antifungal therapy for several months, mortality can be reduced dramatically.
